# On the Utility of Decoction of Rhatany in Keratitis

**Published:** 1855-12

**Authors:** 


On the Utility of Decoction of Rhatany in Keratitis.—Dr. A. Quadri observes, that of all the inflammations of the eye, keratitis is one of the most frequent and most obstinate. Experience has proscribed the employment of mineral astringents. Among those of the vegetable kingdom the laudanum formed by the combination of crocus and opium sometimes produces excellent effects; but in scrofulous ophthalmia, which is frequently but a keratitis, it occasionally gives rise to prolonged and mischievous irritation. The author had tried various other substances, as tannin, calumba, etc., without any definite results, when he resorted to rhatany. The experience of six years has convinced him of its value. Its application merely induces a sensation of dryness in the interior of the eye, and in a short time the pain and photophobia are mitigated, and the weeping is much diminished. When the irritation has thus become calmed in two or three days, the rhatany may be replaced by the more powerful laudanum, more or less diluted. The rhatany is insufficient in the corneitis accompanying blennorrhœal ophthalmia, but in scrofulous and all other forms of keratitis its efficacy is constant. It is prepared by boiling half an oz. of the root in twelve ozs. of water, or decoction of elder-flowers, down to half the quantity, and filtering. It should be freshly prepared, and may be used three or four times a day.—(*Annales d'Oculistique*, tome xxxiii. 87.) *Medico-Chir. Review.*

